# Supplementary figures and images for: The placement of foot-mounted IMU sensors does affect the accuracy of spatial parameters during regular walking
Source: PLoS One. 2022 Jun 9;17(6):e0269567. doi: 10.1371/journal.pone.0269567 (PMC9182246; doi:10.1371/journal.pone.0269567)

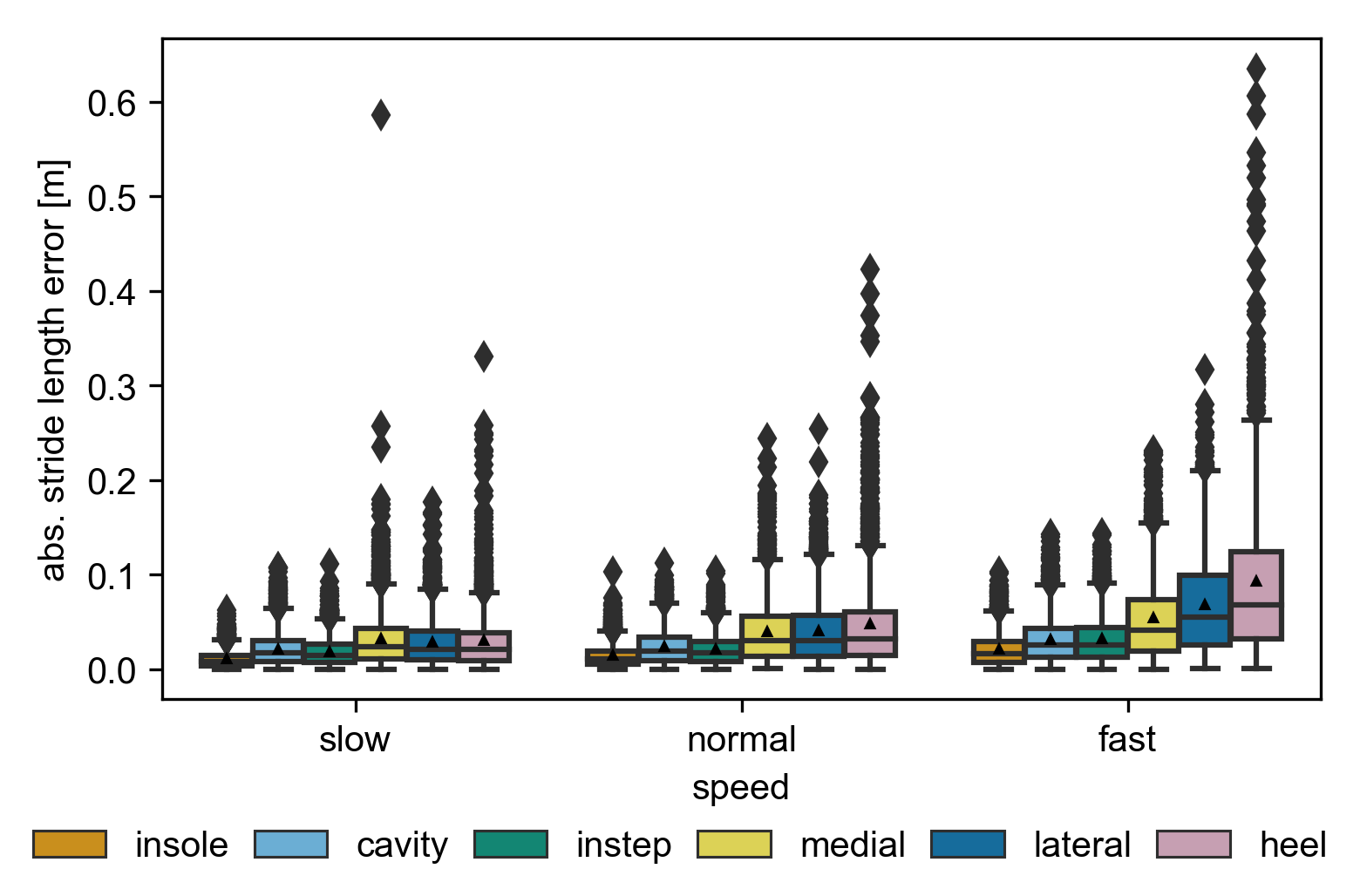

Supplement: S1 Fig — The absolute stride length error over all sensors including all outliers (compare Fig 6). (TIF) [file pone.0269567.s001.tif]

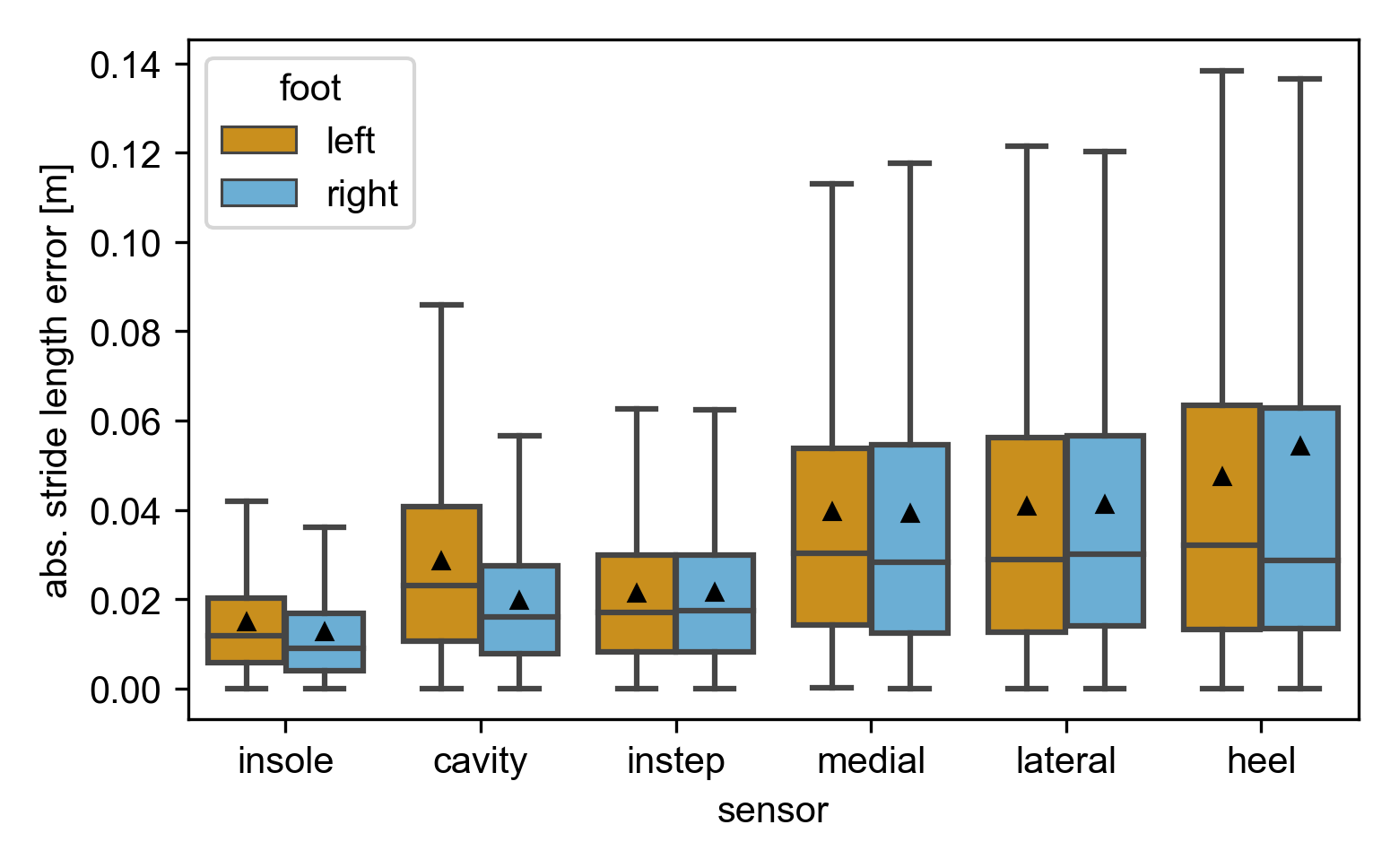

Supplement: S2 Fig — To check if the obtained results are consistent, we calculated the average stride length errors for each foot independently. The figure shows the absolute stride length error over all strides compared between the left and the right foot. The black triangles mark the means of the distributions. All outliers (> Q3 + 1.5IQR) are removed from the visualization. (TIF) [file pone.0269567.s002.tif]

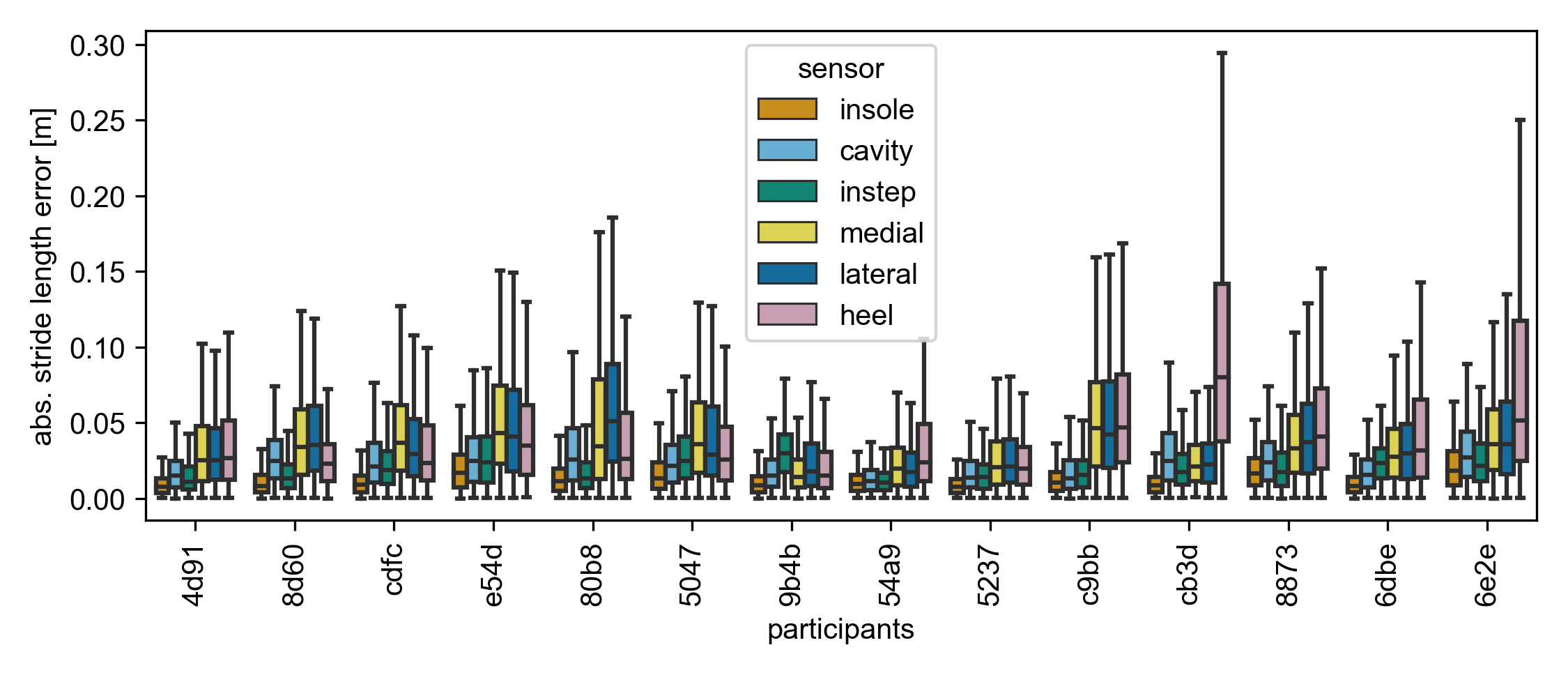

Supplement: S3 Fig — To check if the obtained results are consistent, we calculated the average stride length errors for each participant independently. The figure shows the absolute stride length error over all strides compared over all participants. All outliers (> Q3 + 1.5IQR) are removed from the visualization. The participant id 6dbe actually refers to the recording 6dbe_2 in the published dataset. (TIF) [file pone.0269567.s003.tif]

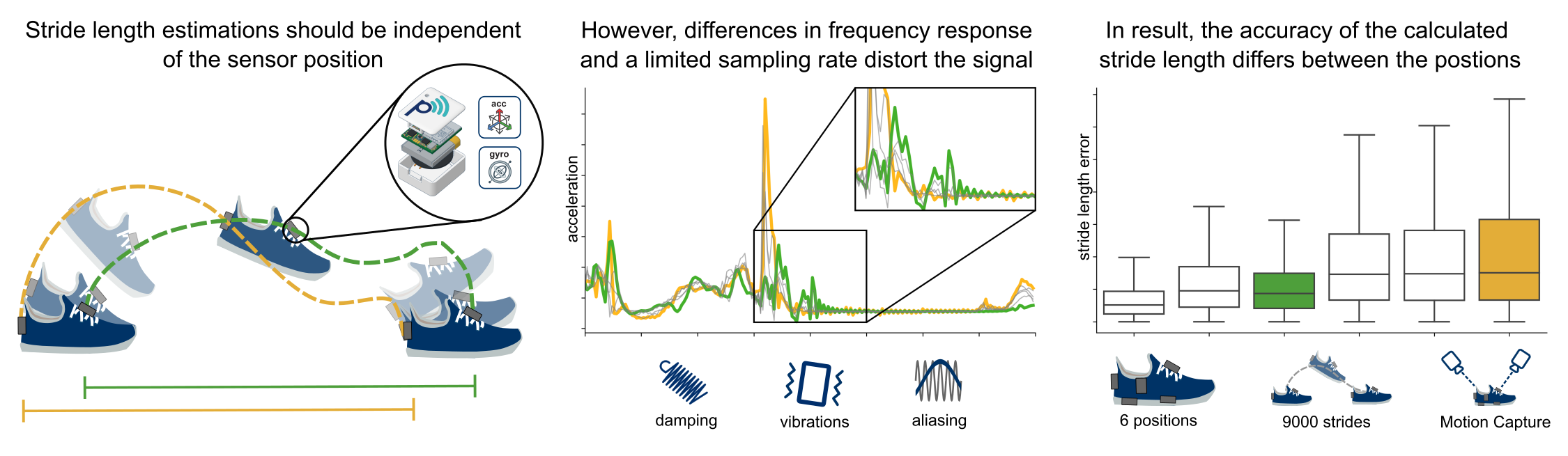

Supplement: S1 Graphical abstract — (TIF) [file pone.0269567.s004.tif]
