# Supplementary material for: The placement of foot-mounted IMU sensors does affect the accuracy of spatial parameters during regular walking
Source: PLoS One. 2022 Jun 9;17(6):e0269567. doi: 10.1371/journal.pone.0269567 (PMC9182246; doi:10.1371/journal.pone.0269567)
Supplement: Attachment — Submitted filename: Response to Reviewers.pdf [file pone.0269567.s005.pdf]

MACHINE LEARNING & DATA ANALYTICS

FAU FRIEDRICH-ALEXANDER UNIVERSITÄT ERLANGEN-NÜRNBERG
TECHNISCHE FAKULTÄT

Responses to the reviewers of the manuscript *The placement of foot-mounted IMU sensors does affect the accuracy of spatial parameters during regular walking (PONE-D-21-39063)*

We would like to thank the editor and reviewer for their time and their positive and constructive feedback. We revised the manuscript accordingly and provided a version of the document with all changes highlighted. Responses to the individual comments are provided below.

Response to the Academic Editor

We adapted the naming of the supplementary files and the main manuscript file. With that, we hope that we now comply with all the requirements of the PLOS One submission guidelines.

Response to the Reviewer

1. *The background introduction and the idea of applying the existing devices to understand how the placement might affect the accuracy are exciting and practical. The purposes of the research are clear and applicable in many fields.*
   Thank you again, for the positive feedback. This is much appreciated.

2. *In the paragraph of "dataset," the unit of gravity should use "G" instead of "g" (Line 115)*

   In this context g refers to "multiples of the gravitational field strength" which is commonly used as an alternative to the official SI unit m/s^2 for acceleration. To the best of our knowledge, using small g is correct in this context. Large G is usually used to denote the general gravitational constant (not earth specific), which we do not mean here. To make this clearer to future readers, we added the range in m/s^2 to the text as well.

3. *In the procedure part of the "dataset" (Line 148-163), "... a continuous 5 min-walk along a path shaped like an eight within a 20 x 5 m area." Please describe if the participants walk in clockwise or counterclockwise. Or the participants could choose whatever they like? The purpose of this is that it might affect the walking pattern and might alter the medial or later placement IMU.*

   We now specify that participants started the eight by walking the first turn counterclockwise. However, we specifically chose the shape of an eight, to avoid issues with left and right turns. With the used shape all participants walked the same duration on a curvilinear path bending to their right and their left.

4. *The data processing is sound, and the analysis of the data seems reasonable. However, the inclusion of other data (the residual energy, the peak acceleration, and PSD) might not be clear for readers in the following discussions. For instance, the main parameter of this research is stride length, which is the distance of the same foot at the minimum velocity at the closest different time points. Even though the maximum acceleration might occur after the initial contact, the correlation of the acceleration and the stride length was not clear enough for readers to understand. Similar to the PSD that higher spectral power in the high-frequency bin does not directly mean that sampling issues will occur. Please highlight the value of those parameters related to the placement of the IMU or the main parameters of strides.*

   While the stride length was the main parameter analyzed in the paper, as this is the most important value from an application perspective. The additional parameters were analyzed to better understand how the sensor position influences the signal in general. With that we hope to contribute to a better understanding of the measurement chain. In addition, the aim was to use these additional parameters to try to explain the origins of the observed stride length error. As we show in the paper, this was unfortunately not really possible and none of the measured parameters does correlate well with the observed error. We still think reporting this process is relevant to create a basis of future research. To make this clearer in the manuscript, we edited the introductory paragraphs in the *Methods / Raw data analysis* and the *Discussion / Raw data analysis* to better explain the intent and aim of this analysis. We hope this makes our analysis easier to understand for future readers.

5. *If there is no significant difference between different conditions, please also provide analysis results that would help the readers understand better during the following discussion.*

Unfortunately, we are not sure what the reviewer means with “different conditions” and therefore can not address this issue directly. However, we agree that some parts of the discussion were not easy to understand. Therefore, we carefully reworked in particular the transitions between the different sections to make it easier to follow the overall story. We hope this resolves this issue and if not, we kindly ask the reviewer to raise it again with more details in a potential next round of reviews.

We want to thank the reviewer again for the time and feedback that has significantly improved the quality of our manuscript.
